# Brunneroma: A Case of Uncommon Gastrointestinal Bleeding in a Young Adult

**DOI:** 10.7759/cureus.35127

**Published:** 2023-02-17

**Authors:** Bassem Al Hariri, Vajeeha Haider, Abdulqadir J Nashwan, Mohamed Mohamedali

**Affiliations:** 1 Internal Medicine, Hamad Medical Corporation, Doha, QAT; 2 Nursing, Hamad Medical Corporation, Doha, QAT

**Keywords:** tumor, gi oncology, young adult, gastrointestinal bleeding, brunner’s gland hyperplasia

## Abstract

Brunner's gland hyperplasia (BGH) or Brunneroma is an uncommon benign proliferative lesion of the small intestine. It is primarily found in the duodenal submucosa, and its main function is to create an alkaline-based mucus to protect the duodenum from stomach acid. BGH can manifest as hyperplasia or a polypoidal tumor and is often discovered incidentally during endoscopy or imaging. Less than 200 cases have been reported in the literature, and it rarely causes gastrointestinal bleeding. In this case report, we present a case of a 25-year-old male who presented with bloody stools and fainting due to severe anemia and underlying gastrointestinal pathology.

## Introduction

Although the small bowel makes up 75% of the digestive tract, tumors of this region are exceedingly uncommon (5%), with duodenal pathology being more prevalent than jejunal and ileal tumors [1]. It is extremely rare to get gastrointestinal bleeding due to Brunner's gland hyperplasia (BGH). It can manifest from hyperplasia to a polypoidal tumor characterized as a Brunner's gland adenoma or hamartoma, respectively [2]. Ten percent of all benign duodenal tumors are BGH, an uncommon benign proliferative lesion with an estimated prevalence of 0.008% [1]. The primary physiological purpose of Brunner's gland, which is situated in the duodenal submucosa, is to create an alkaline-based mucus to shield the duodenal mucosa from the acid released in the stomach [3]. Less than 200 cases have been reported, and it has seldom been discussed in the literature [4]. BGH is mainly discovered incidentally during endoscopy or imaging techniques. Thus, a duodenal polyp or mass must be histopathologically examined for a specific diagnosis and course of treatment [1-2]. We report a case of a young 25-year-old male who presented with the complaint of bloody stools and an episode of fainting secondary to severe anemia and underlying gastrointestinal pathology.

## Case presentation

A 25-year-old male presented in the emergency department with a complaint of dizziness, an episode of fainting, and blood in his stool for the past one week. He mentioned the stools to be black initially, followed by fresh blood mixed with the stool. He had never had these symptoms before. He did not report any hematemesis or epigastric pain. He denied any use of medications or drugs. He was a nonsmoker and nonalcoholic. He had no past medical or surgical history. On examination, the patient looked pale with no abdominal pain. The digital rectal examination was positive for melena, but no fresh blood was seen. The laboratory investigations revealed his hemoglobin to be 6.3 (Table 1).

**Table 1:** Laboratory investigations at admission. White Blood Cells (WBC), Red Blood Cells (RBC), Hemoglobin (Hgb), Hematocrit (Hct), Mean Corpuscular Volume (MCV), Mean Corpuscular Hemoglobin (MCH), Mean Corpuscular Hemoglobin Concentration (MCHC), Red Cell Distribution Width-Coefficient of Variation (RDW-CV), Platelets (PLT), Mean Platelet Volume (MPV), Platelet Distribution Width (PDW), Absolute Neutrophil Count (ANC), Reticulocyte Number (Retic #), Reticulocyte Percentage (Retic %), Prothrombin Time (PT), International Normalized Ratio (INR), Aptt, Urea, Creatinine, Sodium, Potassium, Chloride, Bicarbonate, Bilirubin Total, Protein, Albumin, Alkaline Phosphatase (ALK Phos), Alanine Aminotransferase (ALT), Aspartate Aminotransferase (AST), Troponin-T, High Sensitivity C-Reactive Protein (HS CRP).

Parameters	Results	Reference Range
WBC	11.3	4.0 – 10.0 x10^3/uL
RBC	2.1	4.5 – 5.5 x10^6/uL
Hgb	6.3	13.0 – 17.0 gm/dL
Hct	18.6	40.0 – 50.0 %
MCV	88.2	83.0 – 101.0 fL
MCH	29.9	27.0 – 37.0 pg
MCHC	33.9	31.5 – 34.5 gm/dL
RDW-CV	13.9	11.6 – 14.0 %
Platelets	342	150 – 450 x10^3/uL
MPV	9.3	9.7 – 13.2 fL
PDW	9.0	9.4 -10.6 fL
Absolute Neutrophil count Auto # (ANC)	6.0	2.0 – 7.0 x10^3/uL
Lymphocytes Auto #	4.4	1.0 – 3.0 x10^3/uL
Monocytes Auto#	0.6	0.2 – 1.0 x10^3/uL
Eosinophil Auto#	0.26	0.02 – 0.50 x10^3/uL
Basophils Auto#	0.04	0.02 – 0.10 x10^3/uL
Neutrophil Auto %	53.1	50 – 70 %
Lymphocyte Auto %	38.8	20 – 40 %
Monocyte Auto %	5.4	0.0 – 15.0 %
Eosinophil Auto %	2.3	0.0 – 6.0 %
Basophil Auto %	0.4	0.0 – 2.0 %
Retic #	153.7	50.0 – 100.0 x10^3/uL
Retic %	7.2	0.5 – 2.5 %
Prothrombin time	13.7	9.4 – 12.5 seconds
INR	1.2	1.0 – 1.2
Apt*	29.4	29.1 – 36.5 seconds
Urea	3.8	2.5 – 7.8 mmol/L
Creatinine	92	62 – 106 umol/L
Sodium	141	133 – 146 mmol/L
Potassium	3.7	3.5 – 5.3 mmol/L
Chloride	1.7	95 – 108 mmol/L
Bicarbonate	28	22 – 29 mmol/L
Bilirubin T	6	0 – 21 umol/L
Total Protein	63	60 – 80 gm/L
Albumin	30	35 – 50 gm/L
ALK phosphate	70	40 – 129 U/L
ALT	20	0 – 41 U/L
AST	20	0 – 40 U/L
Troponin-T HS	*8	3 – 15 ng/L
CRP	*3.3	0.0 – 5.0 mg/L

**Figure 1:**
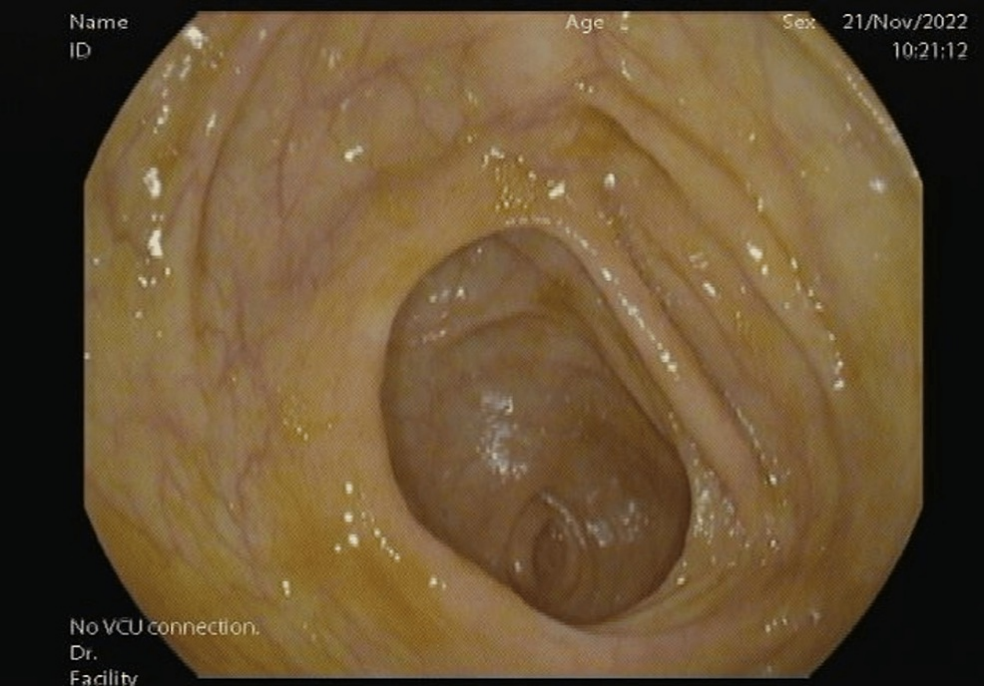
Normal colonic mucosa under colonoscopy; no evidence of bleeding.

He required a blood transfusion of 1 unit. He was vitally stable with a blood pressure of 120/72, a heart rate of 81, a temperature of 37.5 °C, and a respiratory rate of 18. He was referred to Gastroenterology for colonoscopy and esophagogastroduodenoscopy. The colonoscopy was unremarkable for any lower gastrointestinal bleeding as the whole of the colonic mucosa was normal (Figure 1).

**Figure 2:**
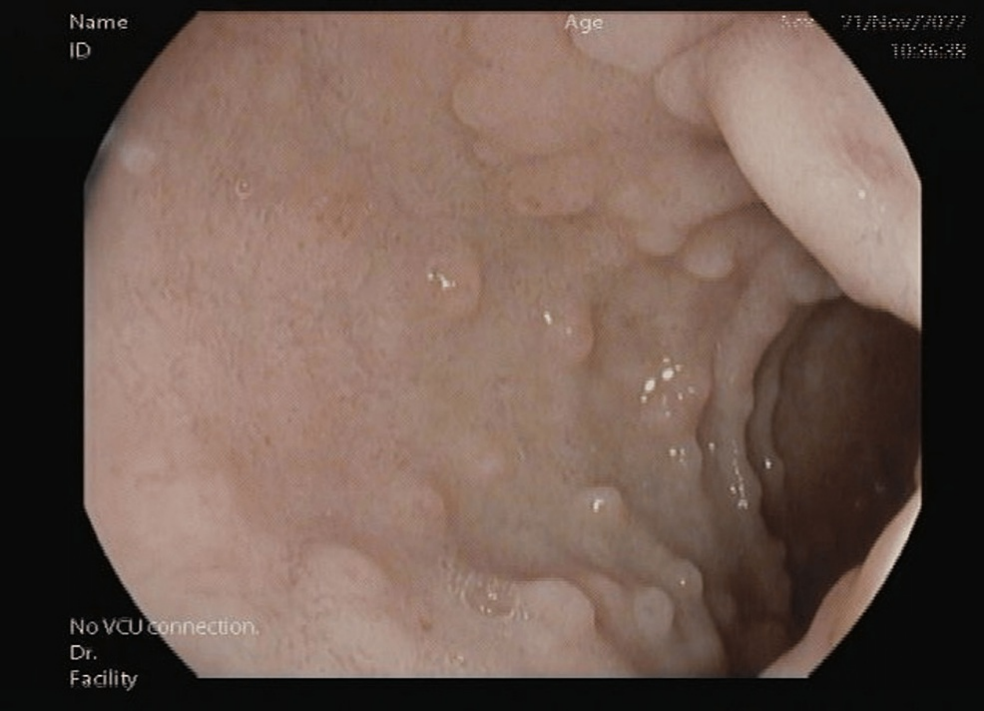
D1 mucosa showing nodularity with no signs of active bleeding.

**Figure 3:**
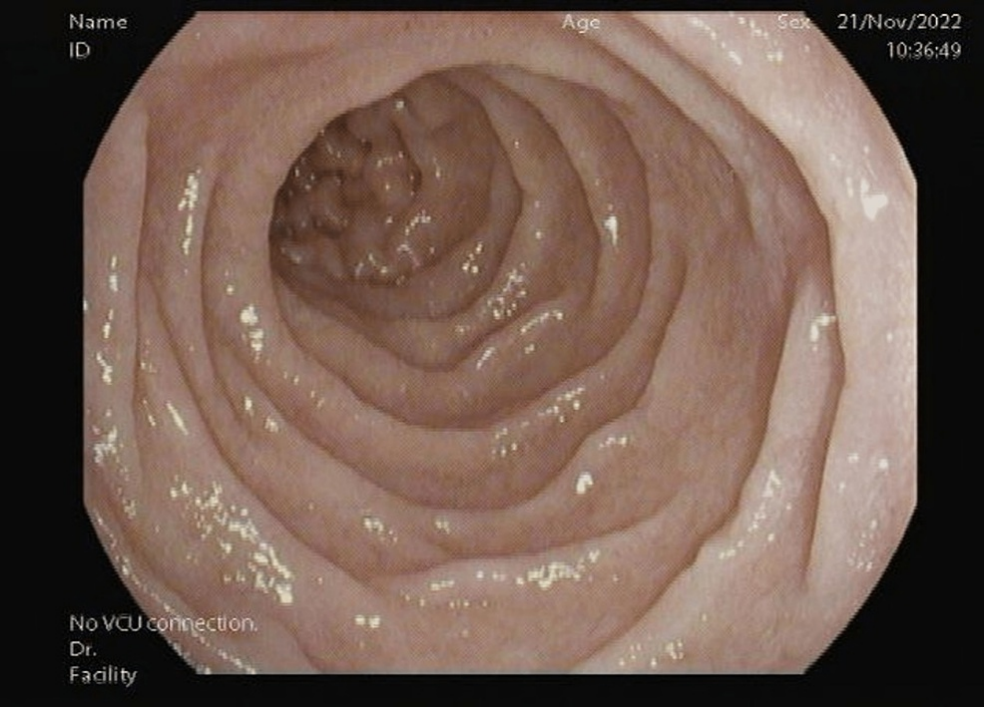
D2 mucosa normal with no nodularity, mass, or bleeding.

Esophagogastroduodenoscopy showed duodenal D1 nodularity, suspecting it to be BGH (Figure 2). Esophagogastroduodenoscopy was negative for any ulcers or lesions in the esophagus and parts of the stomach, such as the fundus, body, or antrum. The biopsies were taken for histopathological examination. D2 showed normal mucosa (Figure 3).

The histopathology biopsy results of the nodules demonstrated small bowel mucosa with BGH, although negative for villous atrophy, crypt hyperplasia, dysplasia, or malignancy. He was therefore treated with supportive measures. Conservative management was opted for as the nodular lesions were multiple and less than 5 mm (2-3 mm). Esmoprazole, 40 mg BID, is administered IV as a 5-day inpatient. During the hospital course, he did not develop further bleeding episodes such as melena or hematemesis. Upon discharge, he has prescribed pantoprazole 40 mg for 3 months. The follow-up for 90 days was uneventful. His hemoglobin also improved.

## Discussion

The American Institute of Radiologic Pathology refers to lesions that are less than 5 mm in size as BGH, while those that are more than 5mm as "Brunner's gland hamartoma" [1]. The literature review says that it’s quite uncommon to get upper gastrointestinal bleeding from isolated Brunner gland hyperplasia. According to histology, BGH is a single or multiple nodular lesion comprising enlarged Brunner's glands divided by fibrous septa. The isolated mass known as Brunner's gland hamartoma comprises a variety of Brunner's glands, ducts, smooth muscle, fibrous tissue, adipose tissue, lymphocytes, etc [1,2,5,6]. These tubulomucinous glands release an alkaline mucin that shields the mucosa of the duodenum from an acidic pH. These are mainly located in the proximal portion of the duodenum's mucosa and submucosa. BGH makes up 3.9%-10% of all benign duodenal polyps and masses. The patients are usually in their 5th or 6th decade with no gender predominance [1,2,6,7]. Though, this patient was relatively young.

The underlying etiology of BGH is unknown. Numerous articles and studies have hypothesized that BGH results from excessive acid secretion, Helicobacter pylori infection, or inflammation encouraging the production of alkaline secretions by the Brunner's gland cells [1-4]. The reduced pancreatic exocrine function may also be the cause. However, it is now believed that the primary component causing BGH is the exocrine modifying factor, which includes the hormone, vagus nerve, and intestinal mucous membrane factor [3,4].

The clinical presentation of Brunner Gland hyperplasia is usually asymptomatic and found incidentally in the elderly. The symptomatic presentation occurs in terms of gastrointestinal bleeding as hematemesis or melena. However, there have been about 15 cases reported for acute hemorrhage leading to hypovolemic shock [5-7].

If BGH is discovered incidentally, it is controversial to treat and requires further research [8]. There are no proper reviews on the medical management of BGH, particularly the smaller diffuse lesions (<5 mm). However, proton pump inhibitors are prescribed to reduce gastric acid secretion if found to be the etiology [3]. All polypoidal masses larger than 2 cm in size, whether symptomatic or not, require treatment. Endoscopic snare-polypectomy or surgical excision is the current treatment option [8].

Regarding our case, there wasn’t any clear evidence of a known underlying cause or previous clinical presentation related to BGH's diagnosis.

## Conclusions

The conclusion that can be drawn from this case report is that BGH should be considered a potential cause of upper gastrointestinal bleeding in adolescents, even though it is a rare condition. This case report highlights the importance of considering rare causes of upper gastrointestinal bleeding in young adults, as it can lead to severe anemia and melena. Healthcare providers should be aware of the potential for rare causes of upper gastrointestinal bleeding in young adults and consider BGH a potential diagnosis. Further research is needed to better understand the prevalence and clinical presentation of BGH in adolescents.
